# Serum metabolomics identifies novel prognostic biomarkers in *amanita* poisoning

**DOI:** 10.3389/fphar.2025.1716911

**Published:** 2025-12-10

**Authors:** Dan Zhu, Jie Zhong, Yarong Liu, Sicheng Zhang, Lianhong Zou

**Affiliations:** 1 The First Affiliated Hospital of Hunan Normal University (Hunan Provincial People’s Hospital), Changsha, Hunan, China; 2 Hunan Provincial People’s Hospital (The First Affiliated Hospital of Hunan Normal University), Changsha, Hunan, China; 3 Institute of Clinical and Translational Medicine, Hunan Provincial People’s Hospital (The First Affiliated Hospital of Hunan Normal University), Changsha, Hunan, China

**Keywords:** mushroom poisoning, biomarkers, mortality risk, serum metabolomics, amatoxins, UPLC-QTOF-MS/MS

## Abstract

**Background:**

*Amanita* poisoning causes 90%–95% of global mushroom-related deaths, yet early prognostic biomarkers for *Amanita* poisoning are lacking.

**Methods:**

33 patients with *Amanita* poisoning were recruited and categorized into survival and death group. Multivariate logistic regression analysis was used to investigate the independent mortality risk factors for *Amanita* poisoning patients. Untargeted serum metabolomics was performed to screen the differentially expressed metabolites. The quality control samples were used to evaluate the stability and reproducibility of ultra performance liquid chromatography quadrupole time-of-flight mass spectrometry (UPLC-QTOF-MS/MS) analytical system. The prognosis predictive metabolic biomarkers were identified by ROC curve analysis. Correlations between metabolic biomarkers and biochemical indicators were analyzed by Spearman’s correlation analysis.

**Results:**

Significant differences were observed between the survival and death groups in clinical manifestations—such as gastrointestinal bleeding, dizziness, headache, delirious coma, infection, and shortness of breath—and in biochemical indicators, including alanine transaminase (ALT), aspartate transaminase (AST), prothrombin time and activated partial thromboplastin time (APTT). Metabolomic analysis identified 80 differentially expressed metabolites involved primarily in amino acid and unsaturated fatty acid metabolism. ROC analysis (AUC >0.9) screened nine potential metabolic biomarkers for predicting clinical outcomes: 9,10-Epoxyoctadecenoic acid, Phosphatidylinositol(16:0/18:2 (9Z,12Z)), N-Acetyl-L-aspartic acid, PI(20:3 (5Z,8Z,11Z)/18:0), Propionylcarnitine, Proline betaine, 4′-Methyl-(−)-epigallocatechin 3-(4-methyl-gallate), PG (18:1 (11Z)/22:6 (4Z,7Z,10Z,13Z,16Z,19Z)), and L-Proline. Notably, correlation analysis revealed that 9,10-Epoxyoctadecenoic acid was positively correlated with AST and activated partial thromboplastin time, whereas 4′-Methyl-(−)-epigallocatechin 3-(4-methyl-gallate), N-acetyl-L-aspartic acid, PI(16:0/18:2 (9Z,12Z)), PI(20:3 (5Z,8Z,11Z)/18:0), and Propionylcarnitine showed negative correlations with various liver and coagulation parameters.

**Conclusion:**

Serum metabolomics has identified metabolic biomarkers capable of predicting mortality in *Amanita* poisoning, with significant correlation to liver and coagulation injury. These biomarkers may facilitate early risk stratification and guide targeted therapeutic interventions. Limitations include small sample size and single-center retrospective design, which may restrict result generalizability.

## 1 Introduction

Mushroom poisoning is a serious public health problem worldwide. It is estimated that 100-200 people die from mushroom poisoning each year in the United States and Europe (Mengs et al., 2012). Investigation through the Foodborne Disease Outbreak Surveillance System from 2010 to 2020 shows that 38676 cases and 788 deaths which were related to mushroom poisoning were reported in China (Li et al., 2021). *Amanita* phalloides, commonly known as “death cap”, is one of the most toxic mushrooms, and responsible for 90%–95% deaths induced by mushroom poisoning worldwide (Garcia et al., 2015a; Zuker-Herman et al., 2021). Three main group toxins were identified in *Amanita* phalloides: amatoxins, phallotoxins and virotoxins (Kayes and Ho, 2024). Among them, amatoxins, especially α-amatoxin, were considered as the main lethal toxins and responsible for organ injury in humans (Garcia et al., 2015b; Le Daré et al., 2021). Because of its stable physical structure, amatoxins are highly soluble in water and present a great resistant to heat, cold, acid, alkali and enzymes, making them difficult to eliminate via various processes (cooking, freezing, drying and metabolism) (Wieland et al., 2008). After a good absorption of gastrointestinal tract, amatoxins were primarily accumulated in liver via transporters including organic anion transport polypeptide 3 (OAYP1B3) (Letschert et al., 2006) and Na + -Taurocholate Co-transporting Polypeptide (NTCP) (Gundala et al., 2004) located in the cell membrane of hepatocytes, and eliminated by kidney during the first 72h of intoxication (Jaeger et al., 1993). Moreover, amatoxins could be excreted into bile and reabsorbed through the enterohepatic circulation, which might aggravate the hepatocyte injury (Sun et al., 2018).

The classic toxic mechanism of *Amanita* poisoning is that amatoxins could reduce the mRNA and protein synthesis via inhibiting RNA polymerase Ⅱ (RNAP Ⅱ) activities and promoting the degradation of RNA Polymerase II Subunit B1 (Rpb1) (Nguyen et al., 1996; Garcia et al., 2014; Xue et al., 2023). Furthermore, apoptosis, autophagy and oxidative stress were involved in the amatoxins induced liver damage (Chen et al., 2020; Gu et al., 2022; Xue et al., 2023). The clinical symptoms and signs of amatoxins intoxication, also known as phalloides toxic syndrome was classically divided into three phase: the gastrointestinal phase, the latency period, and the hepato-renal phase (Karlson-Stiber and Persson, 2003). The treatment for *Amanita* poisoning mainly includes intensive and supportive therapy, prevention of poison absorption, promotion of amatoxins elimination, use of potential antidotes and liver transplantation (Ye and Liu, 2018; Nieminen and Mustonen, 2020; Xu et al., 2023). Although the clinical management of *Amanita* poisoning is constantly evolving and improving, the prognosis of *Amanita* poisoning remains poor.

The reported fatality rates of *Amanita* poisoning vary widely Several recent studies have shown that the mortality rate of *Amanita* poisoning still ranged from 4.4% to 16% (Liu J. et al., 2020; De Olano et al., 2021; Tan et al., 2022; Lecot et al., 2023). Identification of the potential risk factors to predict the prognosis at an early stage may improve clinical outcomes of *Amanita* poisoning patients. A previous study showed that total bilirubin (TB) and APPT were significantly associated with the mortality of patients with wild mushroom induced acute liver injury (Kim et al., 2017). In addition, it is reported that the peak value of ALT, AST, international normalized ratio (INR), and total serum bilirubin (TSB) are more elevated in *Amanita* poisoning patients with fatal outcomes (Tan et al., 2022; Zhang S. et al., 2024). High INR (>3.61) and plasma ammonia (>95.1 μmol/L) were predictors of the poor outcome of *Amanita* phalloides poisoning (Ye et al., 2021). Biochemical factors such as the increase of AST, ALT, lactate dehydrogenase (LDH), TB, PT, INR, and APTT levels are associated with poor outcome in the *Amanita*-containing mushroom poisoning (Trabulus and Altiparmak, 2011). Although several studies have shown that biochemical indicators such as ALT, AST, TSB, TB, LDH, PT, APTT, INR and plasma ammonia can be used to predict the risk of death from *Amanita* phalloides toxin poisoning, the potential of current biochemical indicators for early prediction of the mortality of patients with *Amanita* phalloides toxin poisoning remains unknown. And the risk factors for predicting the mortality of *Amanita* poisoning is still limited.

Metabolomics has successfully utilized in biomarker screening, disease early diagnosis and characterization of biological pathways. Previous research has applied metabolomics to explore the early diagnosis of patients with amatoxin poisoning (Liu et al., 2023) and to investigate amatoxin - induced liver injury mechanisms (Zheng et al., 2023). However, no metabolomic studies have identified prognostic biomarkers for *Amanita* poisoning outcomes. Herein, we enrolled thirty-three patients with *Amanita* poisoning. Twenty-seven of them survived after the treatment, but six died. By comparing the biochemical indicators and serum metabolites of thirty-three patients with *Amanita* poisoning, new biomarkers were identified that could predict the prognosis of patients with *Amanita* poisoning. These biomarkers may contribute to predict disease course and outcomes in patients.

## 2 Materials and methods

### 2.1 Subjects

The study was a retrospective cohort study that examined thirty-three cases of poisoning due to the consumption of *Amanita* phalloides. These cases were admitted to the Department of Emergency Medicine, Hunan Provincial People’s Hospital (the First Affiliated Hospital of Hunan Normal University) in Changsha, China, from July 2017 to October 2020. The diagnosis of *Amanita phalloides* poisoning is most commonly made by a trained mycologist upon examining the mushroom itself or photographs provided. Alternatively, for a subset of cases, laboratory confirmation is achieved by detecting amatoxins via LC-MS/MS in patient samples such as vomitus, blood, urine or the suspected food items. Patients who experienced food poisoning from sources other than *Amanita* phalloides, as well as those with cancers, autoimmune disorders, severe infections, trauma, recent surgeries, or kidney and liver diseases were excluded from the analysis. The follow-up time was calculated from the first day of hospitalization to the date of discharge from hospital or death within 30 days. Based on the clinical outcomes, *Amanita* poisoning patients were divided into the survival group and mortality group. The study was performed in accordance with the Declaration of Helsinki (revised in Fortaleza, Brazil, 2013) and approved by the Medical Ethics Committee of the Hunan Provincial People’s Hospital (IRB Approval No.: [2024]-10).

### 2.2 The treatment for *amanita* poisoning

In the present study, all *Amanita* poisoning patients received the same treatment regimen. Fluid resuscitation and intensive supportive therapy were performed. Activated charcoals were used to minimize the absorption of amatoxins. Hemoperfusion, hemodialysis or plasmapheresis were performed to eliminate the absorbed amatoxins. Drugs such as acetylcysteine, penicillin G and silymarin were used to resist and reduce the toxicity of amatoxins.

### 2.3 Serum samples collection

The blood samples of *Amanita* poisoning patient were all collected in EDTA-containing tubes for serum isolation on the first day of admission to the emergency ward, within 24 h after *Amanita* poisoning occurred. The samples were allowed to stand at room temperature for 30 min, then centrifuged at 2,500 rpm for 10 min at 4 °C (Liu W. et al., 2020). The supernatant was then collected and stored at −80 °C for subsequent analysis. To evaluate the freeze-thaw and long-term storage stability of serum samples, the protein content in the samples was regularly and randomly sampled and tested.

### 2.4 Untargeted UPLC-MS/MS metabolomics

The serum samples were gently thawed at 4 °C. A 100 μL aliquot of each sample was mixed with 20 μL of an internal standard [L-2-chlorophenylalanine (Merck, Germany) 0.3 mg/mL, dissolved in methanol (Purity: ≥99.9% (GC), HPLC grade, Merck, Germany). The resulting mixture was vortexed for 10 s. Subsequently, 300 μL of a precooled methanol/acetonitrile (Purity:≥99.9% (GC), HPLC grade, Merck, Germany) mixture (v/v, 2:1) was added, and the solution was vortexed for 1 min. This was followed by ultrasonic extraction (10 min) in an ice water bath, static (−20 °C, 30 min), centrifuge (13000 rpm, 4 °C, 10 min), 300 μL supernatant was collected (Liu W. et al., 2020). Finally, 150 μL of the supernatant was transferred into an LC-MS injection vial with a leg liner. To avoid the batch effect, the supernatant samples were randomly placed on the sample loading platform of UPLC for analysis. A quality control sample (QC) was prepared by combining the extraction liquids from all samples in equal volumes, ensuring that the volume of the QC matched that of the individual samples. To test the stability and repeatability of the UPLC-QTOF-MS/MS, one QC sample was inserted for every five samples.

UPLC-QTOF-MS/MS analysis was performed on Ultimate 3000 LC system (Thermo Fisher Scientific, United States) and ESI-QTOF-MSMS (Impact II™, Bruker, Germany). Chromatographic separations were performed at 40 °C on an Acclaim TMRSLC120-C18 column (2.1 mm × 100 mm, 2.2 μm, Thermo Fisher Scientific,United States). The mobile phases consisted of phase A, which was a 0.1% ammonium formate aqueous solution (containing 2 mmol/L ammonium formate, prepared with pure water, Agilent, United States), and phase B, which was acetonitrile (Purity:≥99.9% (GC), HPLC grade, Agilent, United States). The flow rate was maintained at a constant 0.2 mL/min, and the injection volume was 10 μL. The gradient elution conditions were as follows: 0–2 min, 2% B; 2–12 min, 50% B; 12–20 min, 90% B; 20–30 min, 90% B; and 30–60 min, 2% B (Liu W. et al., 2020).

The mass spectrometer was operated in both positive and negative electrospray ionization (ESI) modes. The specific instrument parameters were as follows: the capillary voltage was set to 4.5 kV in positive mode and 3.5 kV in negative mode; the dry gas flow was maintained at 8 L/min, and the gas temperature was 200 °C. The nebulizer pressure was established at 2.0 bar, the fragmentor voltage was set to 500 V, and the scaning mode of Impact II™ QTOF mass spectrometer was full scan (20–1,000 m/z).

### 2.5 Data processing and statistical analysis of serum metabolomics

The serum metabolomics data of the survival group and the death group detected by the instrument were simply analyzed using Metaboscape 3.0 software. After noise reduction, peak detection, extraction, alignment and normalization processing in sequence, the detection data were exported for subsequent analysis. The serum metabolites were identified by comparing their molecular weights, fragment patterns and structural information, to the spectral data of metabolites with the same m/z in the standard database of Bruker Company, and freely available human metabolome database (HMDB, https://hmdb.ca/). The information required to be included in the data exported from Metaboscape 3.0 software was: compound name, chemical formula, molecular weight, retention time (RT), as well as sample name and grouping.

Metabolites were identified by comparing their molecular weights, fragment patterns and structural information, to the spectral data of metabolites with the same m/z in the standard database of Bruker Company, and freely available human metabolome database. Because this was an untargeted acquisition, MS/MS spectra were collected in a data-dependent mode; consequently, features were annotated solely by accurate-mass MS^1^ and retention-time matching to HMDB/Bruker libraries, without confirmatory fragment ions.

The metabolomics data preprocessed by Metaboscape 3.0 software were imported into the software MetaboAnalyst 6.0 for principal component analysis (PCA), partial least squares discriminant analysis (PLS-DA) and orthogonal partial least squares discriminant analysis (OPLS-DA) to achieve an in-depth analysis of the overall distribution of the two groups of serum samples, the metabolic profile characteristics and overall differences of the serum metabolites in the survival group and the death group of *Amanita*-containing mushroom poisoning. According to the OPLS-DA analysis, the variable importance in projection (VIP) value was obtained to represent the contribution value of the different expression metabolites in the comparison between groups.

The metabolomics data were preprocessed using MetaboAnalyst 6.0 software, and then t-test, Fold Change analysis, volcano plot and heat map were performed. Serum different expression metabolites in the survival group and the death group of *Amanita* poisoning patients were screened out with the criteria that the variable VIP value was greater than 1, the fold change (FC) was greater than 2 or less than 0.5, and the adjusted *P* value was less than 0.05. Then, metabolic pathway analysis was carried out using MetaboAnalyst 6.0 software, and metabolic pathways with significant interference were screened out with the criteria that the impact value (Impact) was greater than 0.1 and -lg(*p*) was greater than 0.5. Finally, ROC curve analysis was performed on the serum different expression metabolites in the significantly interfered metabolic pathways, and possible biomarkers were screened out with the criterion that ROC was greater than 0.9. The MetaboAnalystR 4.0 platform (https://dev.metaboanalyst.ca/docs/RTutorial.xhtml) provides full details and documentation for R-package.

### 2.6 Statistical analysis

Continuous variables were presented with mean and standard deviation (SD) and compared with Student’s t-test. Categorical variables were expressed as count and percent quantification, which were compaired with the chi-square test or Fisher’s exact test. The non-parametric Mann-Whitney U test was applied to further confirm the differences between survival and death groups. The Shapiro-Wilk test was used for the verification of normality of data distribution. Spearman’s correlation analysis was conducted to explore the correlation between the levels of metabolic biomarkers and biochemical indicators. *P* value <0.05 was considered as statistical significance. The data were analyzed using the statistical software package SPSS 25.0.

## 3 Result

### 3.1 The clinical characteristics of patients with *amanita* poisoning

A total of thirty-three *Amanita* poisoning patients, who were admitted to Hunan Provincial People’s Hospital (the first Affiliated Hospital of Hunan Normal University) between July 2017 and October 2020, were divided into survival and death groups based on the clinical outcomes. Both the t-test (Table 1) and non-parametric tests (Supplementary Table S1) yielded consistent results, revealing statistically significant differences in biochemical indices—including ALT, AST, PT, and APTT—between the survival and non-survival group. Specially, the levels of ALT, AST, PT and APTT in the death group were 4.97, 7.44, 1.84, 1.78 times higher more than those in the survival group respectively, and effect size analysis (Cohen’s d) further validated their clinical importance (Table. 1). In addition, there were significant differences between the survival and death groups of patients with *Amanita* poisoning in terms of clinical features including gastrointestinal bleeding, dizziness and headache, delirious coma, infection, shortness of breath (Table 2). Effect-size analyses revealed very large odds ratios for the five clinical features that distinguished fatal from non-fatal cases (Table 2; Supplementary Table S2), underscoring the clinical gravity of these manifestations beyond statistical significance. Collectively, the above results indicated that the death patients with *Amanita* poisoning had more serious liver and coagulation function injury.

**TABLE 1:** Biochemical indicators of patients with *Amanita* poisoning.

Characteristic	Total (N = 33)	Survival (n = 27)	Death (n = 6)	t/t’	*P-Value*	Effect size (Cohen’s d)
WBC (×10^9^/L)	9.22 ± 0.57	9.42 ± 3.34	8.29 ± 3.19	0.755	0.456	
HGB (g/L)	168.52 ± 33.01	177.70 ± 208.85	127.17 ± 28.92	0.584	0.563	
PLT (×10^9^/L)	179.88 ± 14.26	184.59 ± 71.34	158.67 ± 125.89	0.695	0.492	
NEUT (%)	77.89 ± 14.18	76.41 ± 14.96	84.55 ± 7.57	−1.285	0.208	
ALT (U/L)	644.38 ± 1,210.45	374.31 ± 693.56	1859.70 ± 2,170.16	−3.052	0.005	1.23 (very large)
AST (U/L)	584.55 ± 1,200.87	269.13 ± 431.35	2003.96 ± 2,303.67	−3.821	0.001	1.44 (very large)
TB (μmol/L)	40.46 ± 69,34	38.10 ± 75.50	51.08 ± 30.99	−0.409	0.685	
ALB (g/L)	51.40 ± 58.18	54.07 ± 64.12	39.37 ± 8.24	0.554	0.584	
UA (μmol/L)	359.93 ± 157.64	369.49 ± 150.80	316.93 ± 195.15	0.733	0.469	
Cr (μmol/L)	213.46 ± 263.58	212.29 ± 275.41	218.69 ± 223.99	−0.053	0.958	
BUN (mg/dL)	10.43 ± 6.57	9.85 ± 6.25	13.03 ± 7.92	−1.076	0.290	
MYO (ng/mL)	216.50 ± 246.38	189.13 ± 251.53	339.65 ± 193.03	−1.372	0.180	
CK (U/L)	289.23 ± 599.84	308.24 ± 660.42	203.65 ± 154.97	0.381	0.706	
CK-MB (ng/mL)	46.94 ± 72.37	46.66 ± 78.80	48.67 ± 35.07	−0.064	0.950	
PT (s)	12.65 ± 5.34	10.96 ± 2.39	20.22 ± 8.28	−5.150	0.000	1.73 (very large)
APTT (s)	31.19 ± 12.36	27.29 ± 6.07	48.71 ± 18.32	−5.147	0.000	0.99 (large)
GLU (mmol/L)	6.71 ± 2.65	6.81 ± 2.18	6.22 ± 4.43	0.493	0.625	
LDL (mmol/L)	2.35 ± 1.05	2.46 ± 1.01	1.79 ± 1.13	1.448	0.158	
HDL (mmol/L)	1.31 ± 0.38	1.35 ± 0.34	1.12 ± 0.54	1.338	0.190	
TCHOL (mmol/L)	4.21 ± 1.18	4.35 ± 1.10	3.52 ± 1.35	1.623	0.115	
TG (mmol/L)	1.22 ± 0.69	1.29 ± 0.61	0.89 ± 0.97	1.310	0.200	

Cohen’s d = (mean_death_–mean_Survivor_)/pooled SD., Thresholds: d ≥ 0.8 = large effect; d ≥ 1.0 = Very large effect. WBC, white blood cells; HGB, hemoglobin; PLT, platelets; NEUT%, the percentage of neutrophile granulocytes; ALT, alanine transaminase; AST, aspartate transaminase; TB, total bilirubin; ALB, albumin; UA, uric acid; Cr, creatinine; BUN, blood urea nitrogen; MYO, myoglobin; CK, creatine kinase; CK-MB, creatine kinase; MB, form; PT, prothrombin time; APTT, activated partial thromboplastin time; GLU, glucose; LDL, low density lipoprotein; HDL, high density lipoprotein; TCHOL, total cholesterol; TG, triglyceride.

**TABLE 2:** Clinical data of *Amanita* poisoning patients.

Characteristic	Total (N = 33)	Survival (n = 27)	Death (n = 6)	*X* ^2^	*P*-value	OR (95% CI)
Gender						
Male [N (%)]	18 (54.5)	15 (55.56)	3 (42.86)	0.002	0.996	
Female [N (%)]	15 (45.45)	12 (44.44)	3 (57.14)			
Age (years)	56.67 ± 1.84	56.30 ± 11.19	58.33 ± 7.92	−0.421	0.677	
Latency period (h)						
<6	5 (15.15)	4 (14.81)	1 (16.67)	−0.113	0.910	
6–24	28 (84.85)	23 (85.19)	5 (83.33)			
Initial treatment time(h)	7.99 ± 1.02	7.39 ± 5.49	10.67 ± 7.31	−1.246	0.222	
Length of hospitalization (d)	7.47 ± 4.66	7.49 ± 4.28	5.5 ± 4.35	0.909	0.370	
Disease history [N (%)]						
Diabetes [N (%)]	2 (6.06)	1 (3.70)	1 (16.67)	1.125	0.289	
Hypertension [N (%)]	7 (21.21)	5 (18.52)	2 (33.33)	0.349	0.554	
Coronary heart disease	1 (3.03)	1 (3.70)	0 (0.00)	0.270	0.603	
Personal history						
Smoking [N (%)]	6 (18.18)	5 (18.52)	1 (16.67)	0.075	0.785	
Drinking [N (%)]	3 (9.09)	3 (11.11)	0 (0.00)	0.873	0.350	
Gastrointestinal symptoms						
Abdominal pain [N (%)]	24 (72.73)	18 (66.67)	6 (100.00)	1.575	0.209	
Nausea and vomiting [N (%)]	23 (69.70)	18 (66.67)	5 (83.33)	0.075	0.785	
Gastrointestinal hemorrhage [N (%)]	7 (21.21)	3 (11.11)	4 (66.67)	7.472	0.006	12.6 (1.87–85.01)
Jaundice [N (%)]	11 (33.33)	7 (25.93)	4 (66.67)	2.653	0.103	
Oliguria and anurohematuria [N (%)]	8 (24.24)	7 (25.93)	1 (16.67)	0.452	0.502	
Palpitations and chest pain [N (%)]	2 (6.06)	2 (7.41)	0 (0.00)	0.560	0.454	
Dizziness and headache [N (%)]	7 (21.21)	2 (7.41)	5 (83.33)	14.477	0.000	19.15 (2.42–151.45)
Delirious coma [N (%)]	3 (9.09)	0 (0.00)	3 (50.00)	12.827	0.000	55 (2.32–1,303.06)
Infection [N (%)]	9 (27.27)	4 (14.81)	5 (83.33)	9.668	0.002	19.15 (2.42–151.45)
Shortness of breath [N (%)]	6 (18.18)	2 (7.41)	4 (66.67)	9.746	0.002	31.8 (3.33–303.99)
Poor appetite [N (%)]	28 (84.85)	22 (81.48)	6 (100.00)	0.270	0.603	

OR, odds ratio; CI, confidence interval.

### 3.2 Serum metabolite profiles of the survival and death groups of *amanita* poisoning patients

**FIGURE 1:**
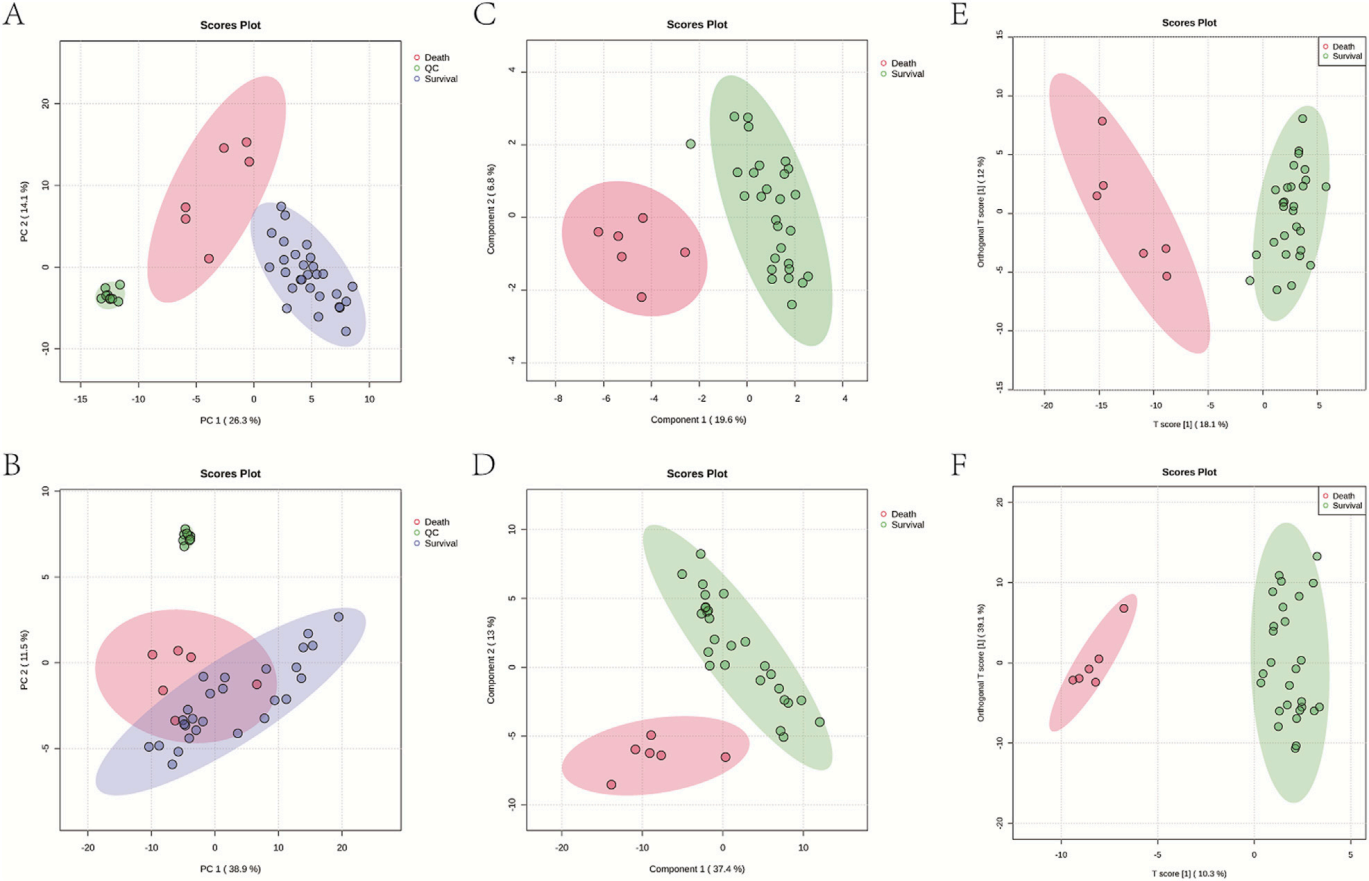
Serum metabolic profile analysis of survival and death groups of patients with *Amanita* poisoning. Plots of PCA **(A)**, PLS-DA **(C)** and OPLS-DA **(E)** scores in the positive ESI mode; Plots of PCA **(B)**, PLS-DA **(D)** and OPLS-DA **(F)** scores in the negative ESI mode.

We performed an untargeted metabolomics analysis using UPLC-QTOF-MS/MS to assess the metabolic differences between the survival and death groups of *Amanita* poisoning patients. The metabolomics data were then analyzed by PCA using MetaboAnalyst 6.0. The position of each sample represents a metabolic profile map (Figures 1A,B). In both positive and negative ESI modes, the serum samples from the survival and death groups were more centrally distributed on the PCA score plot, indicating consistency in the metabolic profiles within the groups. There was a significant separation of the distributions between the two groups, suggesting a significant difference in the metabolic profiles between the survival and death groups of *Amanita* poisoning patients in the positive and negative ESI modes. The sample location of the QC was centralized, indicating stable instrument operation and credible data (Figures 1A,B). PLS-DA analysis showed that serum samples from both the survival and death groups were distributed centrally on the PLS-DA score plots in both positive and negative ESI modes, indicating significant differences between the groups (Figures 1C,D). The metabolomic data of the survival and death groups of *Amanita* poisoning patient were further remodeled using supervised OPLS-DA to obtain the value of the differential authority contribution (VIP value). In both positive and negative ESI models, serum samples from the survival and death groups were differentially distributed on the OPLS-DA score plots (Figures 1E,F). To test the OPLS-DA degree of fitting, the Permutation Test was performed. The explanatory rate (R2Y) was 0.915 and 0.958 in positive and negative ESI models respectively, and the predictive rate (Q2) were 0.746 and 0.775 in positive and negative ESI models respectively, which were greater than 0.5, suggesting that the OPLS-DA model was well fitted and the results of this study were credible (Supplementary Figures 1A,B). The raw serum metabolomics data for patients with Amanita poisoning are provided in Supplementary Data sheet 1, 2.

### 3.3 Screening of serum different expression metabolites in the survival and death groups of *amanita* poisoning patients

**FIGURE 2:**
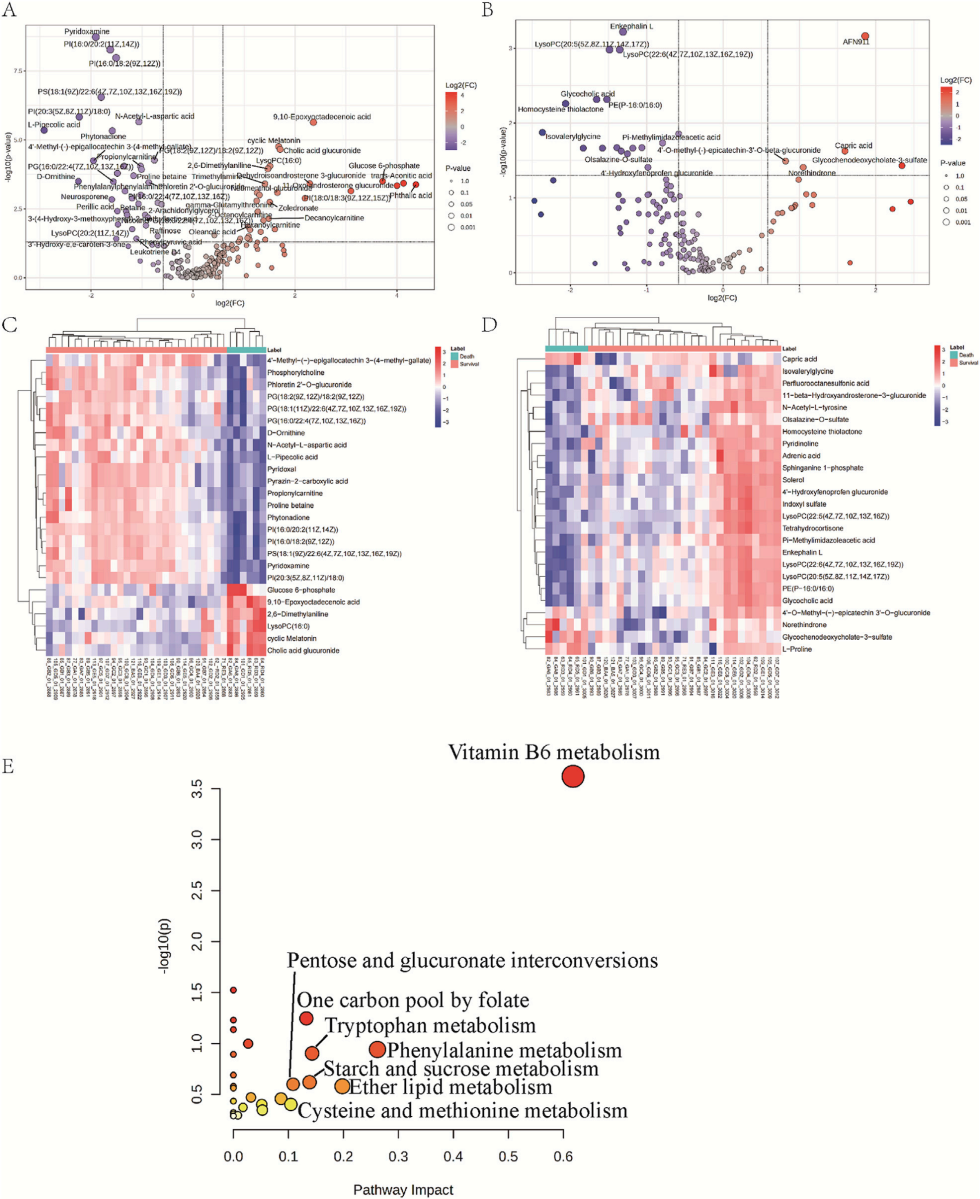
Different expression metabolites analysis and pathways enrichment of surviving and dead patients with *Amanita* poisoning. Volcano plot in the positive **(A)** and negative **(B)** ESI models. Hierarchical clustering heatmaps of the different expression metabolites in the positive **(C)** and negative **(D)** ESI models. Pathway analysis of different expression metabolites between survival and death groups of patients with *Amanita* poisoning **(E)**.

To investigate the different expression metabolites, the Volcanos were analyzed using MetaboAnalyst 6.0 software (Figures 2A,B). 61 and 19 serum different expression metabolites in the positive and negative ESI modes respectively were screened according to the criteria of VIP >1, FC > 2 or <0.5, and *P*. adjusted <0.05 (Supplementary Table 3). Table 3 presents the list of the top 20 differential metabolites based on VIP scores. Subsequently, the significant different expression metabolites were clustered and analyzed in heatmaps, as shown in Figures 2C,D. According to the enrichment analysis, these different expression metabolites were mainly associated with a variety of amino acid metabolism and unsaturated fatty acid metabolism, including vitamin B6 metabolism, phenylalanine metabolism, ether lipid metabolism, tryptophan metabolism, starch and sucrose metabolism, One carbon pool by folate, pentose and glucuronide interconversions and cysteine and methionine metabolism (Figure 2E).

**TABLE 3:** Different expression metabolites between survival and death patients with *Amanita* poisoning.

Metabolites	RT	MW	Formula	FC	Change	VIP	*P* Value	p.Ajusted	ESI
PI(16:0/20:2 (11Z,14Z))	11.82	432.26974	C45H83O13P	0.32	↓	2.14	<0.0001	<0.0001	ESI+
Pyridoxamine	1.05	169.09487	C8H12N2O2	0.27	↓	2.12	<0.0001	<0.0001	ESI+
PI(16:0/18:2 (9Z,12Z))	10.94	418.2538	C43H79O13P	0.35	↓	2.07	<0.0001	<0.0001	ESI+
PS(18:1 (9Z)/22:6 (4Z,7Z,10Z,13Z,16Z,19Z))	8.59	417.75543	C46H76NO10P	0.29	↓	2.01	<0.0001	<0.0001	ESI+
Phytonadione	15.76	473.34452	C31H46O2	0.33	↓	1.95	<0.0001	<0.0001	ESI+
N-Acetyl-L-aspartic acid	1.18	176.04008	C6H9NO5	0.48	↓	1.92	<0.0001	<0.0001	ESI+
L-pipecolic acid	1.02	130.08611	C6H11NO2	0.13	↓	1.89	<0.0001	<0.0001	ESI+
PI(20:3 (5Z,8Z,11Z)/18:0)	8.75	445.2881	C47H85O13P	0.21	↓	1.89	<0.0001	<0.0001	ESI+
Phosphorylcholine	6.37	185.12831	C5H15NO4P	0.50	↓	1.84	<0.0001	<0.0001	ESI+
PG (18:2 (9Z,12Z)/18:2 (9Z,12Z))	9.83	771.47118	C42H75O10P	0.59	↓	1.81	<0.0001	<0.0001	ESI+
PG (18:1 (11Z)/22:6 (4Z,7Z,10Z,13Z,16Z,19Z))	10.54	411.24588	C46H77O10P	0.39	↓	1.79	<0.0001	<0.0001	ESI+
trans-Aconitic acid	1.26	175.03433	C6H6O6	17.49	↑	1.79	<0.0001	<0.0001	ESI+
Enkephalin L	13.46	554.29384	C28H37N5O7	0.40	↓	2.37	<0.0001	0.00060251	ESI-
LysoPC(22:6 (4Z,7Z,10Z,13Z,16Z,19Z))	14.97	566.32221	C30H50NO7P	0.39	↓	2.22	<0.0001	0.0010423	ESI-
LysoPC(20:5 (5Z,8Z,11Z,14Z,17Z))	14.33	540.31545	C28H48NO7P	0.36	↓	2.17	<0.0001	0.0010423	ESI-
PE (P-16:0/16:0)	14.17	436.26744	C21H44NO6P	0.35	↓	2.00	0.000155	0.0048232	ESI-
Glycocholic acid	15.68	464.29968	C26H43NO6	0.32	↓	1.95	0.000178	0.0048232	ESI-
11-Beta-hydroxyandrosterone-3-glucuronide	8.8	481.26211	C25H38O9	0.28	↓	1.83	0.000199	0.021626	ESI-
Perfluorooctanesulfonic acid	9.62	498.91147	C8HF17O3S	0.40	↓	1.81	0.00246	0.023609	ESI-
Pi-methylimidazoleacetic acid	1.08	138.86257	C6H8N2O2	0.58	↓	1.77	0.00113	0.018417	ESI-

### 3.4 Screening the metabolic biomarkers for predicting the mortality risk of patients with *amanita p*oisoning

**FIGURE 3:**
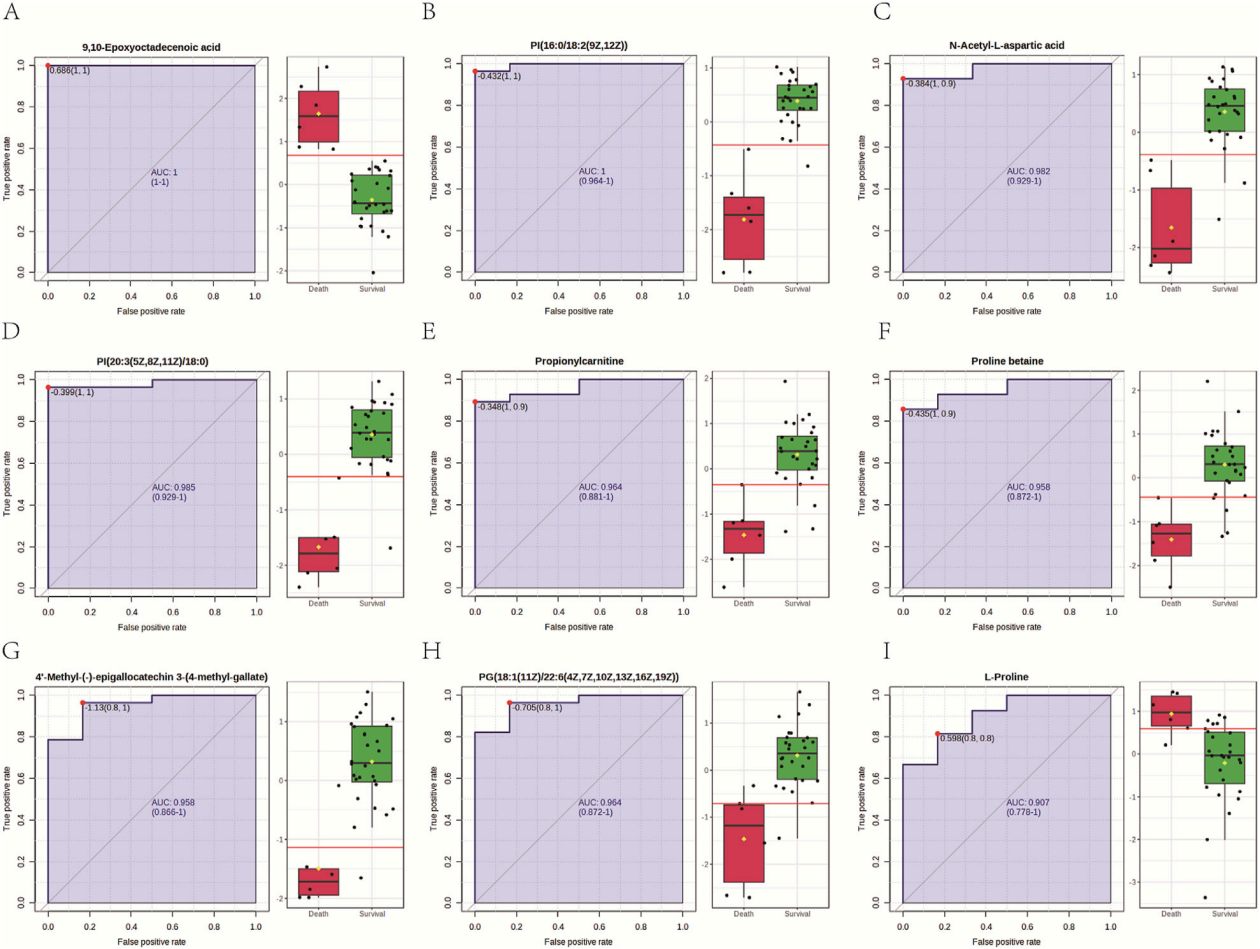
Biomarker screening in survivors and dead patients with *Amanita* poisoning. 9,10-Epoxyoctadecenoic acid **(A)**, PI(16:0/18:2 (9Z,12Z)) **(B)**, N-Acetyl-L-aspartic acid **(C)**, PI(20:3 (5Z,8Z,11Z)/18:0) **(D)**, Propionylcarnitine **(E)**, Proline betaine **(F)**, 4′-Methyl-(−)-epigallocatechin 3-(4-methyl-gallate) **(G)**, PG (18:1 (11Z)/22:6 (4Z,7Z,10Z,13Z,16Z,19Z)) **(H)** and L-Proline **(I)**.

To identify the potential metabolic biomarkers for mortality risk prediction of *Amanita* poisoning patients, the serum different expression metabolites between the survival and death groups of *Amanita* poisoning patients were imported into MetaboAnalyst 6.0 software for ROC curve analysis. Based on the criterion of AUC >0.9, a total of 9 potential biomarkers including 9,10-Epoxyoctadecenoic acid (Figure 3A), PI(16:0/18:2 (9Z,12Z)) (Figure 3B), N-Acetyl-L-aspartic acid (Figure 3C), PI(20:3 (5Z,8Z,11Z)/18:0) (Figure 3D), Propionylcarnitine (Figure 3E), Proline betaine (Figure 3F), 4′-Methyl-(−)-epigallocatechin 3-(4-methyl-gallate) (Figure 3G), PG (18:1 (11Z)/22:6 (4Z,7Z,10Z,13Z,16Z,19Z)) (Figure 3H) and L-Proline (Figure 3I) were screened to predict the clinical outcomes of *Amanita* poisoning patients. The normalized peak intensities of the selected metabolic biomarkers are presented in Figure 3. The optimal cutoff values, determined by the red lines, were used to evaluate the predictive accuracy of these metabolic markers.

### 3.5 Correlation analysis between potential metabolic markers and biochemical indicators of liver and coagulation

**FIGURE 4:**
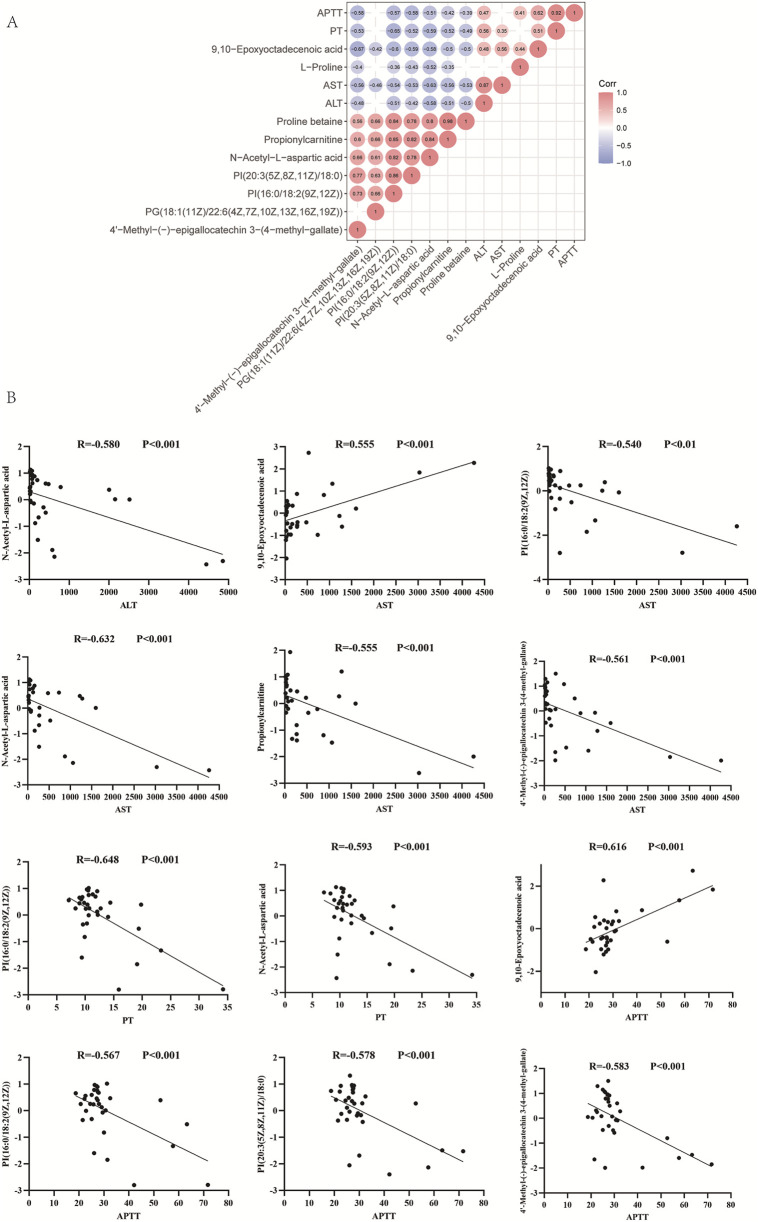
Correlation analysis between potential metabolic markers and biochemical indicators in survival and death groups of *Amanita* poisoning patients. **(A)** The heatmap of correlation coefficients among metabolic biomarkers associated with blood biochemical indicators of liver and coagulation function. The color was corresponded to correlation coefficients with the red representing the positive correlations, and the blue representing the negative correlations. The strength of correlation was represented visually by both numerical percents and circle size. **(B)** Pearson correlations between metabolic markers (N-Acetyl-L-aspartic acid, 9,10-Epoxyoctadecenoic acid, PI(16:0/18:2 (9Z,12Z)), Propionylcarnitine, 4′-Methyl-(−)-epigallocatechin 3-(4-methyl-gallate), PI(20:3 (5Z,8Z,11Z)/18:0)) and serum biochemical indicators of liver and coagulation function (ALT, AST, PT and APPT).

To explore the relationship between metabolic biomarkers and serum biochemical indicators of coagulation and liver function in the survival and death groups of patients with *Amanita* poisoning, correlation analysis was performed. The correlation heat map of potential metabolic markers related to the biochemical indicators of liver and coagulation is shown in Figure 4A. Interestingly, 9,10-Epoxyoctadecenoic acid was positively correlated with AST (Pearson’s *r* = 0.555, *P* < 0.001) and APTT (Pearson’s *r* = 0.616, P < 0.001). 4′-Methyl-(−)-epigallocatechin 3-(4-methyl-gallate) was negatively correlated with AST (Pearson’s *r* = −0.561, *P* < 0.001) and APTT (Pearson’s *r* = −0.583, *P* < 0.001). N-acetyl-L-aspartic acid was negatively correlated with AST (Pearson’s *r* = −0.632, *P* < 0.001), ALT (Pearson’s *r* = −0.580, *P* < 0.001), and PT (Pearson’s *r* = −0.593, *P* < 0.001). PI(16:0/18:2 (9Z,12Z)) was negatively correlated with AST (Pearson’s *r* = −0.540, *P* < 0.001), PT (Pearson’s *r* = −0.648, *P* < 0.001), and APPT (Pearson’s *r* = −0.567, *P* < 0.001). PI(20:3 (5Z,8Z,11Z)/18:0) was negatively correlated with APPT (Pearson’s *r* = −0.578, *P* < 0.001). Propionylcarnitine was negatively correlated with AST (Pearson’s *r* = −0.555, *P* < 0.001). Taken together, these results suggest that the metabolic biomarkers are related to the serum hepatic injury and coagulation dysfunction indicators in *Amanita* poisoning patients (Figure 4B).

## 4 Discussion

The entry of amatoxins into hepatocytes, mediated by the hepatic transporters OATP1B3 and NTCP, initiates lethal liver injury (Wang et al., 2023; Gong et al., 2024). Consequently, the pharmacological inhibition of these transporters presents a viable therapeutic approach for attenuating amatoxin-induced hepatotoxicity (Wang et al., 2023; Xue et al., 2025). This study has obtained a series of results with significant clinical significance through a comprehensive analysis of patients with *Amanita* poisoning from multiple perspectives. Our findings showed that there were significant differences between the survival and death groups of *Amanita* poisoning patients in terms of the clinical features (gastrointestinal bleeding, dizziness and headache, delirious coma, infection, and shortness of breath) and the biochemical indicators (ALT, AST, PT and APTT), suggesting that the death patients with *Amanita* poisoning had more serious liver and coagulation function damage, which were consistent with numerous previous research findings (Trabulus and Altiparmak, 2011; Le Daré et al., 2021). YZ Ye et al. identified the high international normalized ratio (INR) (>3.6) and plasma ammonia (>95.1 μmol/L) as predictive biomarkers of poor prognosis for *Amanita* poisoning patients (Ye et al., 2021). The previous study showed that hepatic encephalopathy (HE), upper gastrointestinal bleeding (UGB), TB concentration, indirect/direct bilirubin ratio, AST, PT, and APTT were significantly associated with the mortality risk factors of *Amanita* poisoning patients (Liu et al., 2023), which was consistent with our study.

Metabolomics analysis provides a novel perspective for revealing the pathophysiological mechanism of *Amanita* poisoning (Liu W. et al., 2020; Liu et al., 2023). In both positive and negative ESI mode, PCA, PLS-DA, and OPLS-DA analyses all demonstrated significant differences in the metabolic characteristics of serum samples between the survival and death groups. A total of 120 serum different expression metabolites were identified, primarily involving multiple amino acid metabolism and unsaturated fatty acid metabolism-related pathways. Vitamin B6 metabolism is the process the body uses to break down and utilize vitamin B6. The primary forms of vitamin B6 in the body include pyridoxal (PL), pyridoxal phosphate (PLP), pyridoxine (PN) and pyridoxamine (PM), as well as their 5′-phosphate esters, with pyridoxal 5′-phosphate (PLP) being the most important and active form. The metabolism of vitamin B6 begins with its absorption in the small intestine. After absorption, vitamin B6 is transported to the liver, where it is converted into its active form, PLP. PLP then acts as a coenzyme for various enzymes involved in amino acid, carbohydrate and lipid metabolism, neurotransmitter synthesis and heme production (Stach et al., 2021). In the present study, Pyridoxamine 5′-phosphate, Pyridoxal and Pyridoxamine were downregulated in the death group of *Amanita* poisoning. When the dysfunction of liver induced by *Amanita* poisoning decreased the conversion of active form Pyridoxamine 5′-phosphate, Pyridoxal and Pyridoxamine from vitamin B6. Therefore, the liver injury caused by *Amanita* poisoning can cause abnormal vitamin B6 metabolism. In addition, the abnormal metabolism of taurine and hypotaurine may interfere with the osmotic pressure regulation and antioxidant function of cells, exacerbating cell damage (Marcinkiewicz and Kontny, 2014); the change in cysteine and methionine metabolism may affect the synthesis of glutathione, weakening the antioxidant defense ability of the body (Martínez et al., 2017). Several studies have indicated a connection between microbial tryptophan metabolism and liver injury (Chen et al., 2025; Ding et al., 2025; Tu et al., 2025). Furthermore, a serum metabolomics study conducted on an α-Amanitin-induced liver injury animal model demonstrated the involvement of the tryptophan metabolism pathway in the hepatotoxic process (Zheng et al., 2023), which aligns with our findings. These consistent results suggest that disrupted tryptophan metabolism may be a key mechanism underlying liver injury in amatoxin poisoning and could represent a potential therapeutic target. These abnormal metabolic pathways are interrelated and interact with each other, jointly constituting the complex metabolic network disorder after *Amanita* poisoning, which may play a key role in the development of the disease.

9,10-Epoxyoctadecenoic acid, PI(16:0/18:2 (9Z,12Z)), N-Acetyl-L-aspartic acid, PI(20:3 (5Z,8Z,11Z)/18:0), Propionylcarnitine, Proline betaine, 4′-Methyl-(−)-epigallocatechin 3-(4-methyl-gallate), PG (18:1 (11Z)/22:6 (4Z,7Z,10Z,13Z,16Z,19Z)) and L-Proline were identified as potential metabolic biomarkers for predicting the mortality risk of *Amanita* poisoning patients. The correlation analysis between these biomarkers and serum biochemical indicators of liver and coagulation functions provided deeper insights into the poisoning mechanism. PI(16:0/18:2 (9Z,12Z)), and PI(20:3 (5Z,8Z,11Z)/18:0) belong to Phosphatidylinositol (PI), which are one of the components of cell membrane and involved in a variety of intracellular signal transduction processes (Porta and Figlin, 2009). During coagulation, PI provides the phospholipid surface required for the conversion of prothrombin to thrombin, which is essential for the blood coagulation process (Liu and McCoy, 1975). Consistent with this, our results showed that the levels of PI(16:0/18:2 (9Z,12Z)) and PI(20:3 (5Z,8Z,11Z)/18:0) were correlated with the PT and APTT. N-Acetyl-L-aspartic acid (NAA) is an amino acid derivative known to be highly abundant in the central nervous system, which is synthesized form aspartic acid and acetyl-CoA by N-acetyltransferase-8-like (NAT8L) and breaked down into aspartate and acetate by aspartoacylase (ASPA) (Krause and Wegner, 2024). Accumulating independently evidences reveals that reduction of N-Acetyl-L-aspartic acid was correlation with acute and chronic central nervous system injury (Niddam et al., 2018; Shibasaki et al., 2018; Li et al., 2020; Hu et al., 2024) and involved in inflammation (Krause and Wegner, 2024). Additional study showed acute liver failure which associated with the poor outcome always accompanied by complication such as hepatic encephalopathy, infection and coagulopathy (Rovegno et al., 2019). Hepatic encephalopathy and septic shock can also cause a degree of central nervous system injury. In the present study, we revealed that the death patients with *Amanita* poisoning had more serious liver injury and infection. Moreover, the level of N-Acetyl-L-aspartic acid is decreased in the death group. Furthermore, the correlation analysis showed that NAA negatively associated with the ALT, AST, PT and APTT in the *Amanita* poisoning patients. Collectively, these results suggested that NAA could serve as a potential indicator of liver damage and coagulopathy, which were associated with the unfavorable prognosis of *Amanita* poisoning. 9,10-Epoxyoctadecenoic acid (9,10-EOA) is a proliferator-activated receptors (PPAR) gamma2 ligand (Lecka-Czernik et al., 2002). An accumulating body of research indicates that PPARgamma/ligand system plays a critical role in regulation of liver regeneration and involves in non-alcoholic fatty liver disease (Yamamoto et al., 2008; Cheng et al., 2018; Zhang L. et al., 2024). In the present study, our results showed that 9,10-Epoxyoctadecenoic acid was upregulated in the death group of *Amanita* poisoning patients and associated with liver injury. These results indicated that 9,10-Epoxyoctadecenoic acid was involved in *Amanita* induced liver damage. However, the specific mechanism by which 10-Epoxyoctadecenoic acid is involved in *Amanita*-induced liver injury requires further experimental confirmation and in-depth exploration. Propionylcarnitine is a derivative of the propionic acid, which is involved in the conversion of fatty acids to energy and thus plays an important role in the energy metabolism. Furthermore, Propionylcarnitine may affect coagulation by improving vascular endothelial function and reducing inflammatory response. Collectively, routine metabolomic screening in suspected cases could expedite hemodialysis or liver transplant decisions.

## 5 Limitations

This study still has some limitations. Firstly, the sample size of this study is small, which may affect the generalizability of the results. Moreover, small sample size limits statistical power. Future multi-center studies with larger cohorts are needed to validate these biomarkers. Secondly, the study is a single-center retrospective study and selection bias inevitably exists. Further validation of these experimental results should be pursued through future multi-center studies. Thirdly, we lacked data on ingested dose, mushroom dry weight, or precise time-to-treatment—variables known to influence outcome. Diabetes, hypertension and coronary disease were evenly distributed between groups (Table 2) and were therefore unlikely drivers of the metabolic signature, but larger studies should include dose–response curves and adjust for Charlson comorbidity index. In addition, although a series of different expression metabolites and potential metabolic biomarkers have been identified, their exact biological functions and molecular mechanisms in the poisoning process have not been thoroughly studied. Further basic experimental research is needed to clarify these aspects. Furthermore, non-targeted metabolomics may miss low-abundance metabolites, and some metabolite identifications relied solely on the first-order mass spectrometry information without MS/MS fragment ion validation. Thus, some of the identification results need to be validated by targeted analysis.

## 6 Conclusion

In summary, this study has conducted a relatively comprehensive analysis of clinical features and metabolomics in patients with *Amanita* poisoning. The death patients with *Amanita* poisoning had more serious liver and coagulation function injury. 9,10-Epoxyoctadecenoic acid, PI(16:0/18:2 (9Z,12Z)), N-Acetyl-L-aspartic acid, PI(20:3 (5Z,8Z,11Z)/18:0), Propionylcarnitine, Proline betaine, 4′-Methyl-(−)-epigallocatechin 3-(4-methyl-gallate), PG (18:1 (11Z)/22:6 (4Z,7Z,10Z,13Z,16Z,19Z)) and L-Proline were served as potential metabolic biomarkers for predicting the prognosis of *Amanita* poisoning patients, which were correlated with serum biochemical indicators of liver and coagulation functions. The present study provided new metabolic biomarkers for prognosis prediction and contributed to improve the therapeutic effect of *Amanita* poisoning.

## Data Availability

The data presented in the study are deposited in the Metabolights database, accession number MTBLS13397, available at: https://www.ebi.ac.uk/metabolights/editor/MTBLS13397/files?reviewCode=cd1a517d-715b-4a62-929b-7dc9d64cf526.

## References

[B1] Chen X. Shao B. Yu C. Yao Q. Ma P. Li H. (2020). The cyclopeptide -amatoxin induced hepatic injury via the mitochondrial apoptotic pathway associated with oxidative stress. Peptides 129, 170314. 10.1016/j.peptides.2020.170314 32387737

[B2] Chen L. Zu M. Cao Y. Wang Y. Jiang A. Bao S. (2025). Oral plant-derived nanomedicines mitigate acetaminophen-induced liver injury by modulating the gut-liver axis and intestinal microbiota metabolism. Small 21 (31), e2502001. 10.1002/smll.202502001 40511710

[B3] Cheng Z. Liu L. Zhang X. J. Lu M. Wang Y. Assfalg V. (2018). Peroxisome proliferator-activated receptor gamma negatively regulates liver regeneration after partial hepatectomy via the HGF/c-Met/ERK1/2 pathways. Sci. Rep. 8 (1), 11894. 10.1038/s41598-018-30426-5 30089804 PMC6082852

[B4] De Olano J. Wang J. J. Villeneuve E. Gosselin S. Biary R. Su M. K. (2021). Current fatality rate of suspected cyclopeptide mushroom poisoning in the United States. Clin. Toxicol. (Phila) 59 (1), 24–27. 10.1080/15563650.2020.1747624 32237919

[B5] Ding F. F. Zhou N. N. Mao Y. J. Yang J. Limbu S. M. Galindo-Villegas J. (2025). Lactiplantibacillus plantarum attenuate gossypol-induced hepatic lipotoxicity by altering intestinal microbiota for enriching microbial tryptophan metabolites in nile tilapia *Oreochromis niloticus* . Microbiome 13 (1), 180. 10.1186/s40168-025-02172-0 40759977 PMC12323027

[B7] Garcia J. Carvalho A. T. Dourado D. F. Baptista P. de Lourdes Bastos M. Carvalho F. (2014). New *in silico* insights into the inhibition of RNAP II by α-amanitin and the protective effect mediated by effective antidotes. J. Mol. Graph Model 51, 120–127. 10.1016/j.jmgm.2014.05.002 24879323

[B8] Garcia J. Costa V. M. Carvalho A. Baptista P. de Pinho P. G. de Lourdes Bastos M. (2015a). Amanita phalloides poisoning: mechanisms of toxicity and treatment. Food Chem. Toxicol. 86, 41–55. 10.1016/j.fct.2015.09.008 26375431

[B9] Garcia J. Costa V. M. Carvalho A. T. P. Silvestre R. Duarte J. A. Dourado D. F. A. R. (2015b). A breakthrough on Amanita phalloides poisoning: an effective antidotal effect by polymyxin B. Archives Toxicol. 89 (12), 2305–2323. 10.1007/s00204-015-1582-x 26385100

[B10] Gong M. Li Z. Xu H. Ma B. Gao P. Wang L. (2024). Amanitin-induced variable cytotoxicity in various cell lines is mediated by the different expression levels of OATP1B3. Food Chem. Toxicol. 188, 114665. 10.1016/j.fct.2024.114665 38641045

[B11] Gu X. Zhang L. Sun W. Liu K. Xu H. Wu P. (2022). Autophagy promotes α-Amanitin-Induced apoptosis of Hepa1-6 liver cells. Chem. Res. Toxicol. 35 (3), 392–401. 10.1021/acs.chemrestox.1c00297 35175747

[B12] Gundala S. Wells L. D. Milliano M. T. Talkad V. Luxon B. A. Neuschwander-Tetri B. A. (2004). The hepatocellular bile acid transporter ntcp facilitates uptake of the lethal mushroom toxin ? amanitin. Archives Toxicol. 78 (2), 68–73. 10.1007/s00204-003-0527-y 14598021

[B13] Hu J. Zhang M. Zhang Y. Zhuang H. Zhao Y. Li Y. (2024). Neurometabolic topography and associations with cognition in alzheimer's disease: a whole-brain high-resolution 3D MRSI study. Alzheimers Dement. 20 (9), 6407–6422. 10.1002/alz.14137 39073196 PMC11497670

[B14] Jaeger A. Jehl F. Flesch F. Sauder P. Kopferschmitt J. (1993). Kinetics of amatoxins in human poisoning: therapeutic implications. J. Toxicol. Clin. Toxicol. 31 (1), 63–80. 10.3109/15563659309000374 8433416

[B16] Karlson-Stiber C. Persson H. (2003). Cytotoxic fungi--an overview. Toxicon 42 (4), 339–349. 10.1016/s0041-0101(03)00238-1 14505933

[B17] Kayes T. Ho V. (2024). Amanita phalloides-associated liver failure: molecular mechanisms and management. Int. J. Mol. Sci. 25 (23), 13028. 10.3390/ijms252313028 39684738 PMC11640968

[B18] Kim T. Lee D. Lee J. H. Lee Y. S. Oh B. J. Lim K. S. (2017). Predictors of poor outcomes in patients with wild mushroom-induced acute liver injury. World J. Gastroenterol. 23 (7), 1262–1267. 10.3748/wjg.v23.i7.1262 28275306 PMC5323451

[B19] Krause N. Wegner A. (2024). N-acetyl-aspartate metabolism at the interface of cancer, immunity, and neurodegeneration. Curr. Opin. Biotechnol. 85, 103051. 10.1016/j.copbio.2023.103051 38103520

[B20] Le Daré B. Ferron P.-J. Gicquel T. (2021). Toxic effects of amanitins: repurposing toxicities toward new therapeutics. Toxins 13 (6), 417. 10.3390/toxins13060417 34208167 PMC8230822

[B21] Lecka-Czernik B. Moerman E. J. Grant D. F. Lehmann J. M. Manolagas S. C. Jilka R. L. (2002). Divergent effects of selective peroxisome proliferator-activated receptor-gamma 2 ligands on adipocyte versus osteoblast differentiation. Endocrinology 143 (6), 2376–2384. 10.1210/endo.143.6.8834 12021203

[B22] Lecot J. Cellier M. Courtois A. Vodovar D. Le Roux G. Landreau A. (2023). Cyclopeptide mushroom poisoning: a retrospective series of 204 patients. Basic Clin. Pharmacol. Toxicol. 132 (6), 533–542. 10.1111/bcpt.13858 36908014

[B23] Letschert K. Faulstich H. Keller D. Keppler D. (2006). Molecular characterization and inhibition of amanitin uptake into human hepatocytes. Toxicol. Sci. 91 (1), 140–149. 10.1093/toxsci/kfj141 16495352

[B24] Li Y. Wang T. Zhang T. Lin Z. Li Y. Guo R. (2020). Fast high-resolution metabolic imaging of acute stroke with 3D magnetic resonance spectroscopy. Brain 143 (11), 3225–3233. 10.1093/brain/awaa264 33141145 PMC7719019

[B25] Li W. Pires S. M. Liu Z. Liang J. Wang Y. Chen W. (2021). Mushroom poisoning outbreaks - china, 2010-2020. China CDC Wkly. 3 (24), 518–522. 10.46234/ccdcw2021.134 34594925 PMC8393043

[B26] Liu D. T. McCoy L. E. (1975). Phospholipid requirements of tissue thromboplastin in blood coagulation. Thromb. Res. 7 (1), 213–221. 10.1016/0049-38487590137-1 1154339

[B27] Liu J. Chen Y. Gao Y. Walline J. H. Lu X. Yu S. (2020). N-acetylcysteine as a treatment for amatoxin poisoning: a systematic review. Clin. Toxicol. (Phila) 58 (11), 1015–1022. 10.1080/15563650.2020.1784428 32609548

[B28] Liu W. Li S. Wu Y. K. Yan X. Zhu Y. M. Jiang F. Y. (2020). Metabolic profiling of rats poisoned with paraquat and treated with xuebijing using a UPLC-QTOF-MS/MS metabolomics approach. Anal. Methods 12 (37), 4562–4571. 10.1039/d0ay00968g 33001064

[B29] Liu Y. Li S. Feng Y. Zhang Y. Ouyang J. Li S. (2023). Serum metabolomic analyses reveal the potential metabolic biomarkers for prediction of amatoxin poisoning. Toxicon 230, 107153. 10.1016/j.toxicon.2023.107153 37178797

[B30] Marcinkiewicz J. Kontny E. (2014). Taurine and inflammatory diseases. Amino Acids 46 (1), 7–20. 10.1007/s00726-012-1361-4 22810731 PMC3894431

[B31] Martínez Y. Li X. Liu G. Bin P. Yan W. Más D. (2017). The role of methionine on metabolism, oxidative stress, and diseases. Amino Acids 49 (12), 2091–2098. 10.1007/s00726-017-2494-2 28929442

[B32] Mengs U. Pohl R. T. Mitchell T. (2012). Legalon® SIL: the antidote of choice in patients with acute hepatotoxicity from amatoxin poisoning. Curr. Pharm. Biotechnol. 13 (10), 1964–1970. 10.2174/138920112802273353 22352731 PMC3414726

[B33] Nguyen V. T. Giannoni F. Dubois M. F. Seo S. J. Vigneron M. Kédinger C. (1996). *In vivo* degradation of RNA polymerase II largest subunit triggered by alpha-amanitin. Nucleic Acids Res. 24 (15), 2924–2929. 10.1093/nar/24.15.2924 8760875 PMC146057

[B34] Niddam D. M. Lai K. L. Tsai S. Y. Lin Y. R. Chen W. T. Fuh J. L. (2018). Neurochemical changes in the medial wall of the brain in chronic migraine. Brain 141 (2), 377–390. 10.1093/brain/awx331 29236991

[B35] Nieminen P. Mustonen A. M. (2020). Toxic potential of traditionally consumed mushroom Species-A controversial continuum with many unanswered questions. Toxins (Basel) 12 (10), 639. 10.3390/toxins12100639 33023182 PMC7599650

[B36] Porta C. Figlin R. A. (2009). Phosphatidylinositol-3-kinase/Akt signaling pathway and kidney cancer, and the therapeutic potential of phosphatidylinositol-3-kinase/Akt inhibitors. J. Urol. 182 (6), 2569–2577. 10.1016/j.juro.2009.08.085 19836781

[B37] Rovegno M. Vera M. Ruiz A. Benítez C. (2019). Current concepts in acute liver failure. Ann. Hepatol. 18 (4), 543–552. 10.1016/j.aohep.2019.04.008 31126880

[B38] Shibasaki J. Aida N. Morisaki N. Tomiyasu M. Nishi Y. Toyoshima K. (2018). Changes in brain metabolite concentrations after neonatal hypoxic-ischemic encephalopathy. Radiology 288 (3), 840–848. 10.1148/radiol.2018172083 29893645

[B39] Stach K. Stach W. Augoff K. (2021). Vitamin B6 in health and disease. Nutrients 13 (9), 3229. 10.3390/nu13093229 34579110 PMC8467949

[B40] Sun J. Zhang Y.-T. Niu Y.-M. Li H.-J. Yin Y. Zhang Y.-Z. (2018). Effect of biliary drainage on the toxicity and toxicokinetics of Amanita exitialis in beagles. Toxins 10 (6), 215. 10.3390/toxins10060215 29799455 PMC6024615

[B41] Tan J. L. Stam J. van den Berg A. P. van Rheenen P. F. Dekkers B. G. J. Touw D. J. (2022). Amanitin intoxication: effects of therapies on clinical outcomes - a review of 40 years of reported cases. Clin. Toxicol. (Phila) 60 (11), 1251–1265. 10.1080/15563650.2022.2098139 36129244

[B42] Trabulus S. Altiparmak M. R. (2011). Clinical features and outcome of patients with amatoxin-containing mushroom poisoning. Clin. Toxicol. (Phila) 49 (4), 303–310. 10.3109/15563650.2011.565772 21563906

[B43] Tu D. Lu C. Guo J. Chen Q. Li X. Wang Y. (2025). Gut microbiota-mediated berberine metabolism ameliorates cholestatic liver disease by suppressing 5-HT production. Clin. Mol. Hepatol. 10.3350/cmh.2025.0577 41087029 PMC12835802

[B44] Wang B. Wan A. H. Xu Y. Zhang R. X. Zhao B. C. Zhao X. Y. (2023). Identification of indocyanine green as a STT3B inhibitor against mushroom alpha-amanitin cytotoxicity. Nat. Commun. 14 (1), 2241. 10.1038/s41467-023-37714-3 37193694 PMC10188588

[B45] Wieland T. Faulstich H. Fiume L. (2008). Amatoxins, phallotoxins, phallolysin, and antamanide: the biologically active components of poisonous amanita mushrooms. CRC Crit. Rev. Biochem. 5 (3), 185–260. 10.3109/10409237809149870 363352

[B46] Xu Y. Wang S. Leung C. K. Chen H. Wang C. Zhang H. (2023). α-amanitin induces autophagy through AMPK-mTOR-ULK1 signaling pathway in hepatocytes. Toxicol. Lett. 383, 89–97. 10.1016/j.toxlet.2023.06.004 37329965

[B47] Xue J. Lou X. Ning D. Shao R. Chen G. (2023). Mechanism and treatment of α-amanitin poisoning. Arch. Toxicol. 97 (1), 121–131. 10.1007/s00204-022-03396-x 36271256

[B48] Xue J. Lou X. Ning D. Yang Y. Shao R. Liu Y. (2025). Ezetimibe protects against alpha-amanitin-induced hepatotoxicity by targeting the NTCP receptor: mechanistic insights from *in vitro* and *in vivo* models. Toxicon 264, 108423. 10.1016/j.toxicon.2025.108423 40449755

[B49] Yamamoto Y. Ono T. Dhar D. K. Yamanoi A. Tachibana M. Tanaka T. (2008). Role of peroxisome proliferator-activated receptor-gamma (PPARgamma) during liver regeneration in rats. J. Gastroenterol. Hepatol. 23 (6), 930–937. 10.1111/j.1440-1746.2008.05370.x 18565023

[B50] Ye Y. Liu Z. (2018). Management of Amanita phalloides poisoning: a literature review and update. J. Crit. Care 46, 17–22. 10.1016/j.jcrc.2018.03.028 29627659

[B51] Ye Y. Liu Z. Zhao M. (2021). CLIF-OF 9 predicts poor outcome in patients with Amanita phalloides poisoning. Am. J. Emerg. Med. 39, 96–101. 10.1016/j.ajem.2020.01.027 31982218

[B52] Zhang L. Xu L. Rong A. Cui Y. Wang L. Li L. (2024). Effect of Rab18 on liver injury and lipid accumulation by regulating perilipin 2 and peroxisome proliferator-activated receptor gamma in non-alcoholic fatty liver disease. J. Gastroenterol. Hepatol. 39 (10), 2219–2227. 10.1111/jgh.16676 39030773

[B53] Zhang S. Fan M. Zhang Y. Li S. Lu C. Zhou J. (2024). Establishment and validation of a nomogram model for prediction of clinical outcomes in patients with Amanita phalloides poisoning. Heliyon 10 (17), e37320. 10.1016/j.heliyon.2024.e37320 39295998 PMC11409095

[B54] Zheng C. Lv S. Ye J. Zou L. Zhu K. Li H. (2023). Metabolomic insights into the mechanisms of ganoderic acid: protection against α-Amanitin-Induced liver injury. Metabolites 13 (11), 1164. 10.3390/metabo13111164 37999259 PMC10672867

[B55] Zuker-Herman R. Tong R. Wong A. (2021). Intravenous rifampicin use in the management of amanita phalloides toxicity. Clin. Toxicol. 59 (9), 843–845. 10.1080/15563650.2021.1887492 33605821

