# Supplementary material for: Serum metabolomics identifies novel prognostic biomarkers in amanita poisoning
Source: Front Pharmacol. 2025 Dec 10;16:1716911. doi: 10.3389/fphar.2025.1716911 (PMC12728034; doi:10.3389/fphar.2025.1716911)
Supplement: Supplementary file 2 [file Table1.docx]

**Supplementary Table 1.** Biochemical indicators of patients with Amanita poisoning

| Biochemical Parameter | Total (N=33) | Survival (n=27) | Death (n=6) | P-value | Effect size (r) |
| --- | --- | --- | --- | --- | --- |
| WBC (×10⁹/L) | 8.54 (7.25-10.71) | 9.14 (7.53-10.78) | 7.48 (6.63-8.10) | 0.199 | 0.078 |
| HGB (g/L) | 142.00 (123.00-154.00) | 142.00 (125.50-154.00) | 136.50 (115.50-153.75) | 0.744 | 0.020 |
| PLT (×10⁹/L) | 205.00 (153.00-242.00) | 207.00 (171.50-241.50) | 133.00 (100.00-222.25) | 0.294 | 0.063 |
| NEUT (%) | 81.30 (74.00-86.20) | 77.80 (71.75-85.40) | 86.55 (85.05-88.65) | 0.069 | 0.114 |
| ALT (U/L) | 115.30 (29.30-411.50) | 79.50 (24.40-183.50) | 605.15 (453.32-3490.12) | 0.002 | 0.231 |
| AST (U/L) | 126.90 (32.60-529.09) | 63.10 (29.52-210.49) | 749.60 (553.32-1016.44) | 0.005 | 0.202 |
| TB (μmol/L) | 19.30 (14.60-34.80) | 16.60 (13.75-25.05) | 44.40 (29.18-68.18) | 0.021 | 0.152 |
| UA (μmol/L) | 387.30 (247.00-492.30) | 387.30 (280.10-499.35) | 310.50 (147.80-417.70) | 0.441 | 0.046 |
| Cr (μmol/L) | 87.40 (62.70-199.30) | 81.10 (62.20-165.95) | 101.39 (76.38-277.98) | 0.560 | 0.035 |
| BUN (mg/dL) | 8.98 (5.30-12.11) | 8.75 (5.06-11.69) | 12.32 (9.33-12.76) | 0.363 | 0.055 |
| MYO (ng/ml) | 97.10 (48.80-172.20) | 88.30 (47.30-133.60) | 377.90 (198.72-476.75) | 0.005 | 0.202 |
| CK (U/L) | 105.60 (78.00-193.00) | 102.00 (73.95-166.55) | 153.75 (102.35-193.00) | 0.363 | 0.055 |
| CK-MB (ng/mL) | 24.00 (17.00-41.00) | 24.00 (16.50-34.50) | 39.00 (21.00-79.50) | 0.413 | 0.049 |
| PT (s) | 10.70 (10.30-14.00) | 10.60 (10.10-12.15) | 23.30 (20.15-24.72) | <0.001 | 0.311 |
| APTT (s) | 28.00 (26.10-31.40) | 27.30 (25.10-29.35) | 57.70 (46.00-68.20) | 0.001 | 0.252 |
