# Supplementary figures and images for: Serum metabolomics identifies novel prognostic biomarkers in amanita poisoning
Source: Front Pharmacol. 2025 Dec 10;16:1716911. doi: 10.3389/fphar.2025.1716911 (PMC12728034; doi:10.3389/fphar.2025.1716911)

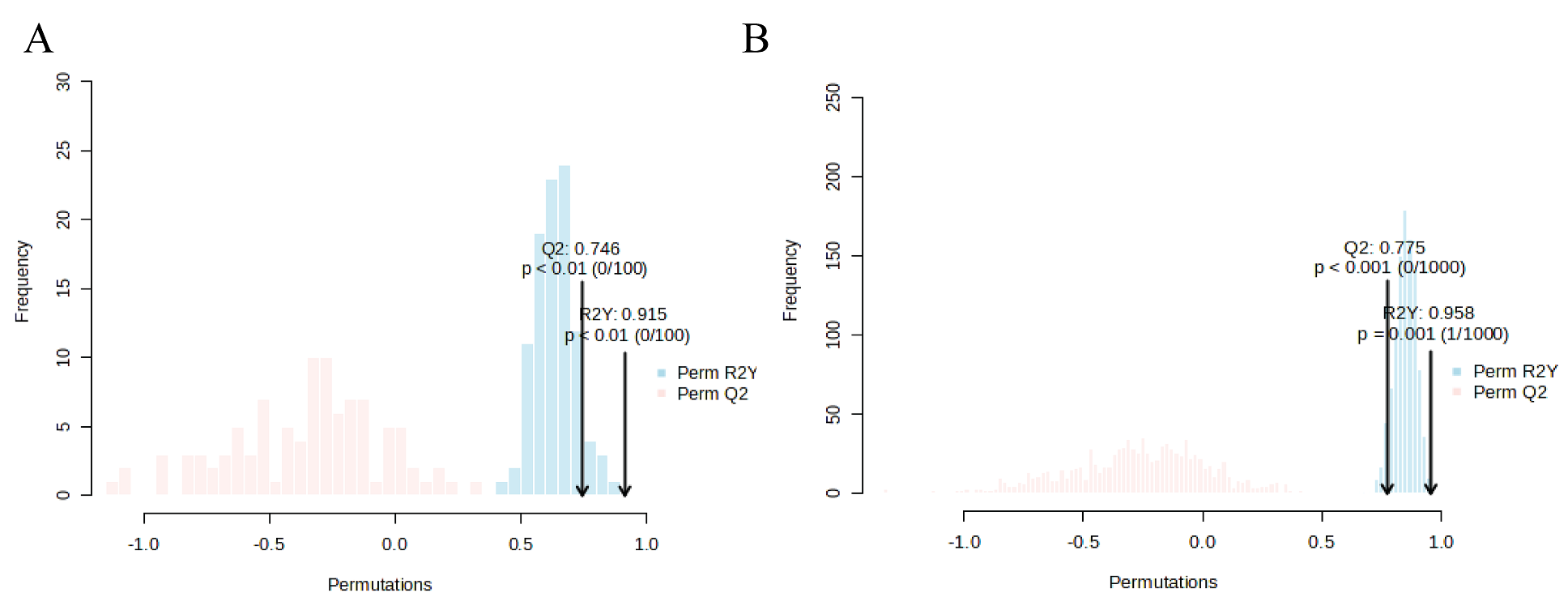

Supplement: Supplementary file 3 [file Image1.tif]
