# Supplementary material for: Serum metabolomics identifies novel prognostic biomarkers in amanita poisoning
Source: Front Pharmacol. 2025 Dec 10;16:1716911. doi: 10.3389/fphar.2025.1716911 (PMC12728034; doi:10.3389/fphar.2025.1716911)
Supplement: Supplementary file 4 [file Table2.docx]

Table 2. Risk Factors for Mortality in Amanita Poisoning Patients (OR Analysis)

| **Characteristic [N(%)]** | **Total** | **Survival (n=27)** | **Death(n=6)** | **OR (95% CI)** | **P-value** |
| --- | --- | --- | --- | --- | --- |
| Male | 18(54.5) | 15(55.56) | 3(42.86) | 1.07 (0.2-5.63) | 1 |
| Latency period (<6h) | 5(15.15) | 4(14.81) | 1(16.67) | 0.78 (0.03-18.42) | 1 |
| Diabetes | 2(6.06) | 1(3.70) | 1(16.67) | 2.78 (0.3-25.72) | 0.464 |
| Hypertension | 7(21.21) | 5(18.52) | 2(33.33) | 1.84 (0.31-10.87) | 0.616 |
| Coronary heart disease | 1(3.03) | 1(3.70) | 0(0.00) | 0.78 (0.03-18.42) | 1 |
| Diabetes | 1(3.03) | 1(3.70) | 0(0.00) | 0.78 (0.03-18.42) | 1 |
| Abdominal pain | 24(72.73) | 18(66.67) | 6(100.00) | 3.18 (0.15-65.37) | 0.556 |
| Nausea and vomiting | 23(69.70) | 18(66.67) | 5(83.33) | 0.9 (0.12-6.84) | 1 |
| Gastrointestinal hemorrhage | 7(21.21) | 3(11.11) | 4(66.67) | 12.6 (1.87-85.01) | 0.011 |
| Jaundice | 11(33.33) | 7(25.93) | 4(66.67) | 4.13 (0.72-23.61) | 0.159 |
| Oliguria and anurohematuria | 8(24.24) | 7(25.93) | 1(16.67) | 0.75 (0.1-5.45) | 1 |
| Palpitations and chest pain | 2(6.06) | 2(7.41) | 0(0) | 0.78 (0.03-18.42) | 1 |
| Dizziness and headache | 7(21.21) | 2(7.41) | 5(83.33) | 19.15 (2.42-151.45) | 0.003 |
| Delirious coma | 3(9.09) | 0(0) | 3(50) | 55 (2.32-1303.06) | 0.004 |
| Infection | 9(27.27) | 4(14.81) | 5(83.33) | 19.15 (2.42-151.45) | 0.003 |
| Shortness of breath | 6(18.18) | 2(7.41) | 4(66.67) | 31.8 (3.33-303.99) | 0.002 |

Note: OR = Odds Ratio; CI = Confidence Interval

Data are presented as n(%) or OR (95% CI)
